# Effects of Nitrogen Availability on the Antioxidant Activity and Carotenoid Content of the Microalgae *Nephroselmis* sp

**DOI:** 10.3390/md18090453

**Published:** 2020-08-29

**Authors:** Noémie Coulombier, Elodie Nicolau, Loïc Le Déan, Vanille Barthelemy, Nathalie Schreiber, Pierre Brun, Nicolas Lebouvier, Thierry Jauffrais

**Affiliations:** 1 ADECAL Technopole, 1 bis rue Berthelot, 98846 Noumea, New Caledonia; 2 Ifremer, RBE/BRM/LPBA, Rue de l’île d’Yeu, 44311 Nantes, France; Elodie.Nicolau@ifremer.fr (E.N.); Nathalie.Schreiber@ifremer.fr (N.S.); 3 Ifremer, IRD, Univ Nouvelle-Calédonie, Univ La Réunion, UMR 9220 ENTROPIE, BP 32078, 98800 Nouméa, New Caledonia; Loic.Le.Dean@ifremer.fr (L.L.D.); Vanille.Barthelemy@ifremer.fr (V.B.); Pierre.Brun@ifremer.fr (P.B.); Thierry.Jauffrais@ifremer.fr (T.J.); 4 ISEA, EA7484, Université de la Nouvelle Calédonie, Campus de Nouville, 98851 Nouméa, New Caledonia; nicolas.lebouvier@univ-nc.nc

**Keywords:** lutein, natural products, nutrients, peroxyl radical, siphonaxanthin

## Abstract

*Nephroselmis* sp. was previously identified as a species of interest for its antioxidant properties owing to its high carotenoid content. In addition, nitrogen availability can impact biomass and specific metabolites’ production of microalgae. To optimize parameters of antioxidant production, *Nephroselmis* sp. was cultivated in batch and continuous culture conditions in stirred closed photobioreactors under different nitrogen conditions (N-repletion, N-limitation, and N-starvation). The aim was to determine the influence of nitrogen availability on the peroxyl radical scavenging activity (oxygen radical absorbance capacity (ORAC) assay) and carotenoid content of *Nephroselmis* sp. Pigment analysis revealed a specific and unusual photosynthetic system with siphonaxanthin-type light harvesting complexes found in primitive green algae, but also high lutein content and xanthophyll cycle pigments (i.e., violaxanthin, antheraxanthin, and zeaxanthin), as observed in most advanced chlorophytes. The results indicated that N-replete conditions enhance carotenoid biosynthesis, which would correspond to a higher antioxidant capacity measured in *Nephroselmis* sp. Indeed, peroxyl radical scavenging activity and total carotenoids were higher under N-replete conditions and decreased sharply under N-limitation or starvation conditions. Considering individual carotenoids, siphonaxanthin, neoxanthin, xanthophyll cycle pigments, and lycopene followed the same trend as total carotenoids, while β-carotene and lutein stayed stable regardless of the nitrogen availability. Carotenoid productivities were also higher under N-replete treatment. The peroxyl radical scavenging activity measured with ORAC assay (63.6 to 154.9 µmol TE g^−1^ DW) and the lutein content (5.22 to 7.97 mg g^−1^ DW) were within the upper ranges of values reported previously for other microalgae. Furthermore, contents of siphonaxanthin ere 6 to 20% higher than in previous identified sources (siphonous green algae). These results highlight the potential of *Nephroselmis* sp. as a source of natural antioxidant and as a pigment of interest.

## 1. Introduction

Microalgae are recognized as promising sources of natural antioxidant products for nutraceuticals, pharmaceuticals, and cosmetics industries [1,2,3,4,5]. Their antioxidant properties are attributed to the large content and variety of molecules, including ascorbic acid, tocopherols, phenolic acids, and carotenoids [6,7,8,9,10]. The antioxidant activity of carotenoids is related to their photoprotective function against photo-oxidative damages caused by reactive oxygen species (ROS), which are continuously produced through photosynthesis and aerobic metabolism [11,12]. Photoprotective carotenoids protect the cell against oxidative stress via several mechanisms including (i) dissipation of excess energy by heat through the xanthophyll cycle [13], (ii) peroxyl radical scavenging (iii), singlet oxygen quenching, and (iv) by preventing the formation of singlet oxygen by deactivating photosensitizers such as triplet-state chlorophyll [14,15,16]. Carotenoids can be divided in two groups: (i) the primary carotenoids pertaining to the photosystems (PSs) with light harvesting and photoprotective function; and (ii) the secondary carotenoids, which do not pertain to the PSs but still have a photoprotective function [17]. Generally, primary carotenoids are more abundant when conditions are favorable for growth, while secondary carotenoids increase in response to stressor action [17]. Under stressful growth conditions such as excess of light, nutrient starvation, high salinity, or extreme temperatures, production of ROS is stimulated [18]. As a defense response to excess ROS, antioxidant molecules, including secondary carotenoids, can accumulate in the cells of some microalgae [19,20,21,22]. Thus, to enhance the production of antioxidants, modification of culture conditions can be implemented to mimic environmental stresses.

Nitrogen availability can impact the production of biomass and specific metabolites. Indeed, as a major component of proteins, nucleic acids, and chlorophylls, nitrogen (N) is one of the most important elements known to influence the biochemical content of microalgae. In addition, N-starvation is known to induce ROS generation in microalgae cells [23,24,25]. Indeed, several authors have shown an increase of hydrogen peroxide [23,25,26] and an increase of lipid peroxidation [23,24,25,27,28] in microalgae cells as a result of N-starvation. ROS increase leads to a deep variation of the antioxidant content [29,30,31,32,33], a well-known example being the massive accumulation of β-carotene in *Dunaliella* spp. exposed to N-starvation combined with high light and high salinity [19,30,34]. However, the influence of nitrogen availability on antioxidant compounds is species-specific, and high antioxidant activity due to over-accumulation of secondary carotenoids under nutrient stress should not be generalized to all species. Indeed, only some chlorophytes species are able to accumulate secondary carotenoids [17], whereas several studies describe a decrease of antioxidant activity and primary carotenoids with N-starvation [25,29,35,36].

In this study, we focus on the effects of nitrogen availability on the antioxidant activity and carotenoid content of *Nephroselmis* sp. It is a chlorophyte species isolated in a tropical lagoon in New Caledonia that was previously identified as a good source of natural antioxidant owing to its high carotenoid content [37]. In addition, *Nephroselmis* sp. contains siphonaxanthin [37,38], an uncommon pigment of interest for biotechnological applications. Indeed, it was shown that this ketocarotenoid, which is mainly found in primitive green algae, has several bioactive properties, including antioxidant activity [39], but also antiangiogenic [40], antiobesity [41,42,43], anti-inflammatory [44], and apoptosis-inducing effect [45].

In a previous study, it was found that high light conditions induced an increase of carotenoid contents, including siphonaxanthin, as well as peroxyl radical scavenging activity on *Nephroselmis* sp. [37]. However, the effects of other culture parameters (e.g., pH, temperature, nutrient) on antioxidant activity and carotenoid contents, especially siphonaxanthin, have not yet been investigated in *Nephroselmis* sp. The aim of this study was to assess the effects of nitrogen availability, combined with high light intensity, on the antioxidant activity and carotenoid content of *Nephroselmis* sp. in order to optimize these factors.

## 2. Results

### 2.1. Identification, Growth, and Cellular Elemental Composition

The phylogenic analysis distributes the microalgal 18S rRNA sequence in the *Nephroselmis* genus (Supplementary Materials Figure S1). The *Nephroselmis* sp. (N3C46) is closely related to *Nephroselmis rotunda* (M0932, [46]), with 93% branch support (Supplementary Materials Figure S1).

**Figure 1:**
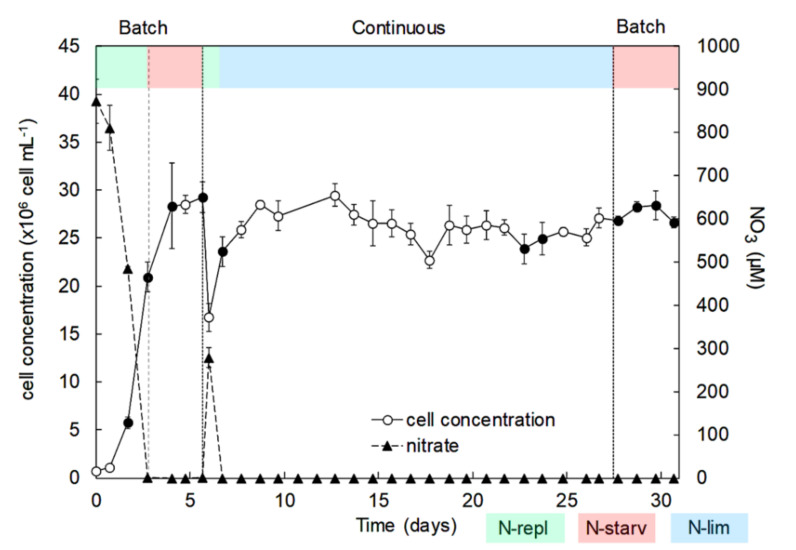
Cell concentrations (cell mL^−1^) and residual nitrate concentrations (µM) over time for *Nephroselmis* sp. cultures in PBRs in batch and continuous modes. Black dots represent sample collection for antioxidant activity and carotenoids analysis. Data are expressed as mean ± standard error (SE, *n* = 2).

To evaluate the impacts of nitrogen availability on the growth and cellular elemental composition of *Nephroselmis* sp., the experiment was separated into 3 successive stages with various nitrogen conditions (Figure 1): (1) a first batch culture period in successive N-replete (N-repl) and N-starvation (N-starv) conditions (batch); (2) a continuous culture in chemostat with N-limitation (continuous N-lim); and (3) a second batch culture period in N-starvation until senescence (batch N-starv).

**Figure 2:**
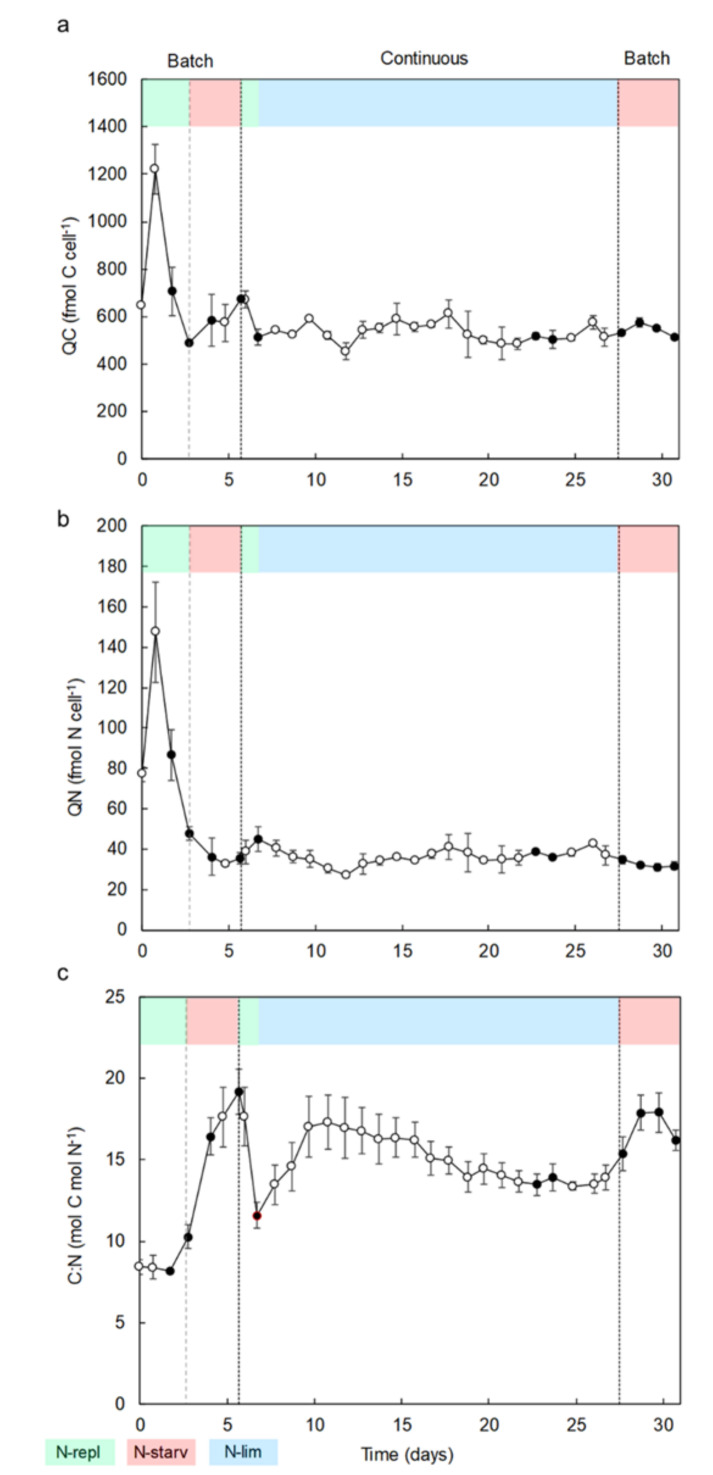
(**a**) Carbon cell quota (QC, fmol cell^−1^). (**b**) Nitrogen cell quota (QN, fmol cell^−1^). (**c**) Carbon/nitrogen (C/N) ratios over time for *Nephroselmis* sp. cultures in PBRs in batch and continuous modes. Black dots represent sample collections for antioxidant activity measures and carotenoid analysis. Data are expressed as mean ± standard error (SE, *n* = 2).

**Figure 3:**
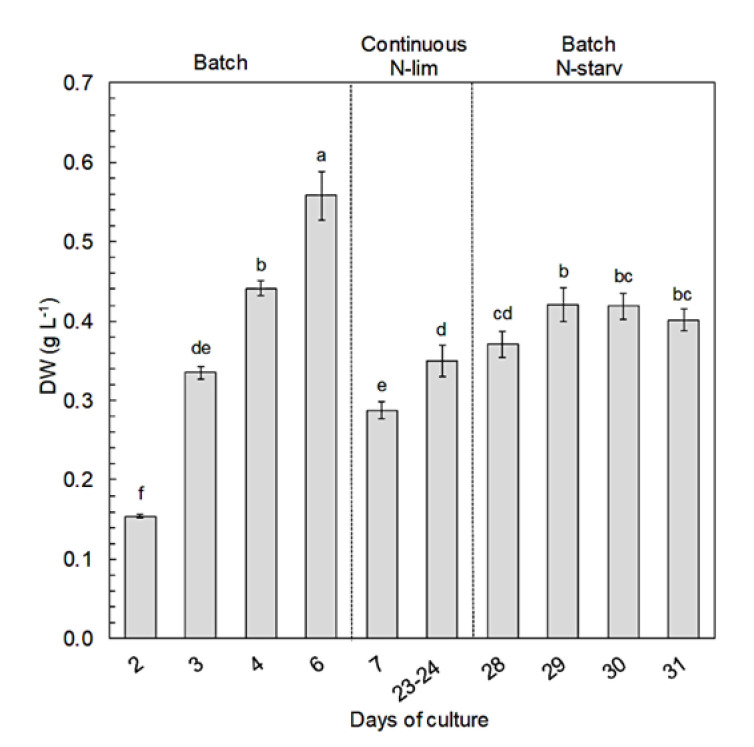
Dry weights (DWs) of Nephroselmis sp. biomass (g L^−1^) at different times of the culture. Data are expressed as mean ± standard error (SE, *n* = 2). Different letters indicate statistically significant differences (*p* < 0.05).

During the first batch culture period (day 0 to day 6), nitrate in culture medium was depleted over three days, however cells kept dividing until day 4, reaching a maximum cell concentration of 29 × 10^6^ ± 0.3 × 10^6^ cell mL^−1^ (Figure 1). N-starvation directly impacted the carbon cellular content, which increased by 38% from day 3 to day 6 (Figure 2a), i.e., cell division stopped but total biomass production (dry weight) still increased significantly until 0.56 g L^−1^ on day 6 (Figure 3). On the contrary, the nitrogen cellular content decreased by 78% from day 1 to its lowest values at the end of the first batch culture period of ~32 fmol N cell^−1^ (Figure 2b). The carbon/nitrogen (C/N) ratio of around 8 under N-replete conditions increased up to 19.2 under N-starvation conditions (Figure 2c). At day 7, the cultures were switched from batch to continuous mode. Then, the photobioreactors (PBRs) refilled with culture medium led to culture dilution and nitrate resupply (Figure 1), which induced cell division, a decrease of cellular carbon content, an increase of cellular nitrogen content, and a drop of the C/N ratio (Figure 2). A steady state was reached at day 20, with a cell concentration of 25 × 10^6^ ± 0.5×10^6^ cell mL^−1^ and a production of biomass of 0.35 ± 0.02 g L^−1^ (Figure 3). Since the culture was N-limited, nitrogen cellular content remained lower (36 ± 0.7 fmol N cell^−1^) and the C/N ratio (14 ± 0.2) remained higher than in N-replete conditions. At day 28, the cultures were switched back to batch, which induced a decrease of the nitrogen cellular content to its lowest values (~32 fmol N cell^−1^), whereas the cell concentration, carbon content, and biomass production increased at the beginning of the N-starvation. The growth and carbon content declined when the senescence phase began. Dissolved phosphate persisted in the medium throughout all experiments (Supplementary Materials Figure S2), indicating no phosphate limitation.

### 2.2. Antioxidant Activity

**Figure 4:**
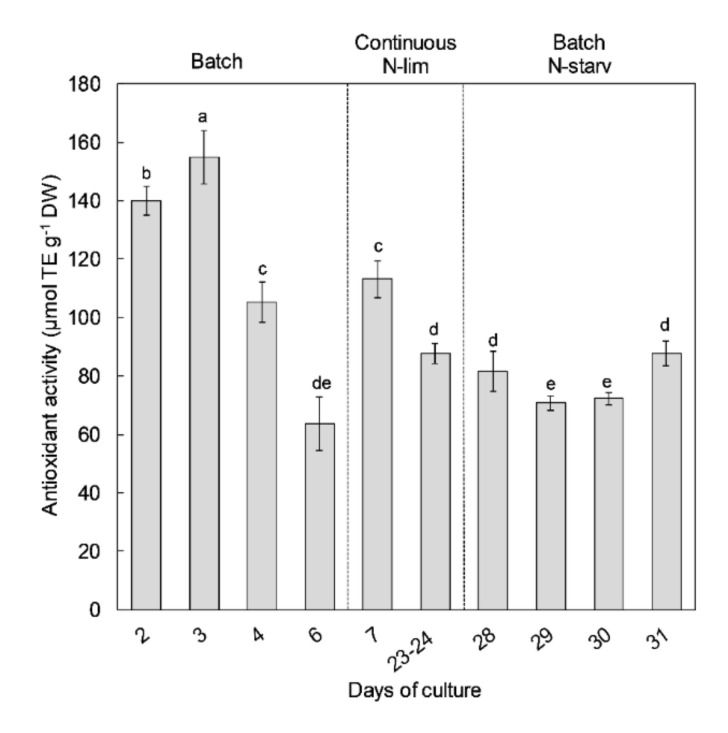
Antioxidant activity measured with ORAC assays (µmol Trolox equivalent g^−1^ DW) of *Nephroselmis* sp. at different times of the culture. Different letters indicate statistically significant differences (*p* < 0.05). Data are expressed as mean ± standard error (SE, *n* = 2).

Antioxidant activity of *Nephroselmis* sp. was measured with ORAC assays at different times of the culture. Antioxidant activity values ranged from 63.6 to 154.9 µmol TE g^−1^ DW (Figure 4). The highest antioxidant activities were found during the exponential growth phase of the first batch culture period at days 2 and 3. The highest value (154.9 µmol TE g^−1^ DW) reached at day 3 was concomitant to the first day of the nitrate starvation in the culture medium. In all conditions with N-limitation or starvation (i.e., steady state in continuous mode or the stationary phases in batch), antioxidant activity was more than 2 times lower than in N-replete cultures. After nutrient resupply on day 7, antioxidant activity increased by 47%, but maximal antioxidant activity observed on day 3 was not restored.

### 2.3. Pigment Content and Composition

**Figure 5:**
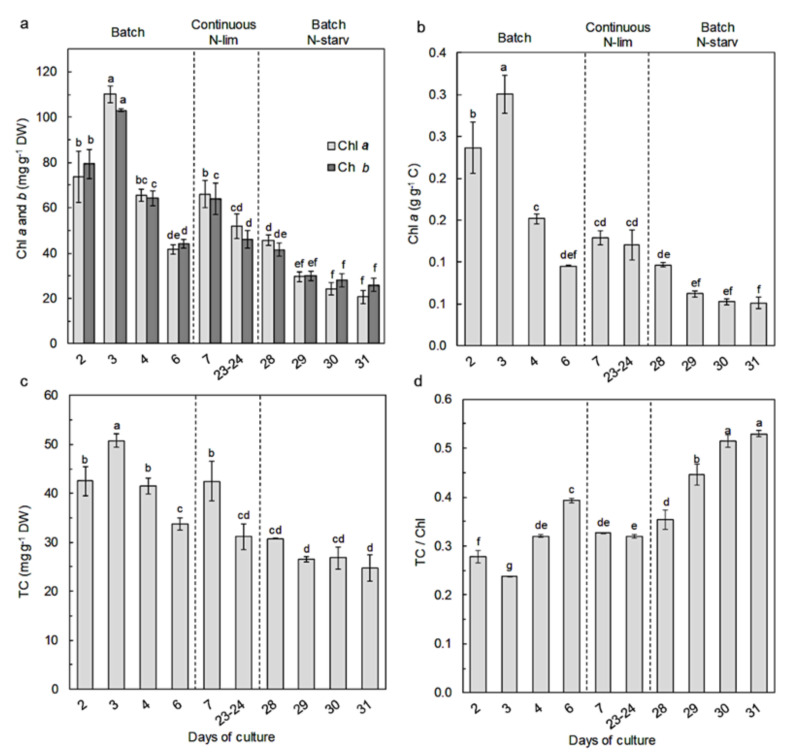
Variations of pigment contents in *Nephroselmis* sp. at different times of the culture: (**a**) contents of chlorophylls *a* and *b* (mg g^−1^ DW); (**b**) chlorophyll *a* content (g g^−1^ carbon); (**c**) total carotenoid (TC) content (mg g^−1^ W); (**d**) total carotenoids/chlorophyll. Data are expressed as mean ± standard error (SE, *n* = 2). Different letters indicate statistically significant differences (*p* < 0.05).

Pigment profiles and content compositions were measured at different experimental stages during the culture. The pigments of *Nephroselmis* sp. include chlorophyll *a* (chl *a*), chlorophyll *b* (chl *b*), β-carotene, lycopene, lutein, zeaxanthin, antheraxanthin, violaxanthin, all-*trans*-neoxanthin, *cis*-neoxanthin, and siphonaxanthin (see HPLC chromatogram in Supplementary Materials Figure S3). Chlorophyll *a* (express on dry weight basis or on carbon content basis), chlorophyll *b*, and total carotenoid (TC) contents (Figure 5a–c) followed similar variations. During the first batch culture period, their contents increased throughout the exponential growth phase and reached the highest concentrations on day 3 (chl *a* 110.1 ± 3.8 mg g^−1^ DW, chl *b* 103.0 ± 0.5 mg g^−1^ DW, TC 50.7 ± 1.3 mg g^−1^ DW), when nitrate was depleted in the culture medium. Once nitrate was depleted, chlorophyll and total carotenoid content quickly decreased from day 3 to day 6 by a factor 2 and by a factor 1.5, respectively. After nutrient resupply (day 7), the chlorophyll and total carotenoid increased by 59% and 26% and then decreased by 22% and 26%, respectively, at steady state. During the second batch period, the pigment content decreased along with the N-starvation, reaching its lowest values (chl *a* 20.69 ± 2.93 mg g^−1^ DW, chl *b* 26.03 ± 2.87 mg g^−1^ DW, TC 24.72 ± 2.78 mg g^−1^ DW) at the end of the experiment. The TC/Chl ratio (Figure 5d) followed opposite variations—during the first batch period, the TC/Chl ratio slightly decreased in the exponential growth phase (day 2 to day 3) and started to increase from 0.24 ± 0.00 to 0.39 ± 0.00 after nitrate starvation. After nutrient resupply, the TC/Chl ratio decreased and reached a similar value as the steady state (~0.33). During the second batch period (day 28), the TC/Chl ratio increased until reaching its highest value at day 31 (0.53 ± 0.01).

**Figure 6:**
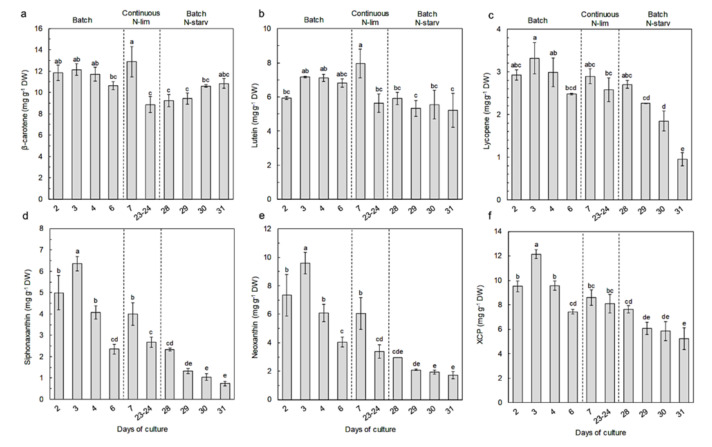
Variations of carotenoids (mg g^−1^ DW) in *Nephroselmis* sp. at different times of the culture: (**a**) β-carotene; (**b**) lutein; (**c**) lycopene; (**d**) siphonaxanthin; (**e**) neoxanthin (*trans* and *cis*); (**f**) xanthophyll cycle pigments (XCP; violaxanthin + antheraxanthin + zeaxanthin). Data are expressed as mean ± standard error (SE, *n* = 2). Different letters indicate statistically significant differences (*p* < 0.05).

Regarding individual carotenoids (Figure 6, Table S1), β-carotene was the major carotenoid, representing 24% to 44% of total carotenoids, followed by lutein, which represented 14 to 21% of the total carotenoids. Their concentrations were stable throughout the experiment, ranging from 8.86 ± 0.74 to 12.9 ± 1.4 mg g^−1^ DW for β-carotene and from 5.22 ± 1.00 to 7.97 ± 0.84 mg g^−1^ DW for lutein.

The other carotenoids were present in smaller concentrations. Siphonaxanthin and neoxanthin varied concomitantly with chlorophyll. Their highest concentration was measured on day 3 (6.36 ± 0.33 and 9.58 ± 0.75 mg g^−1^ DW, respectively), and similarly to chlorophyll, siphonaxanthin and neoxanthin drastically declined along with N-starvation, reaching values of 0.74 ± 0.12 and 1.72 ± 0.25 mg g^−1^ DW, respectively, on day 31. The xanthophyll cycle pigments (i.e., violaxanthin, antheraxanthin, and zeaxanthin) and lycopene followed the same trend as siphonaxanthin and neoxanthin, but with less variations of the concentrations. Their respective concentrations varied from 12.14 ± 0.35 mg g^−1^ DW and 3.32 ± 0.37 mg g^−1^ on day 3 to 5.24 ± 0.88 mg g^−1^ DW and 0.95 ± 0.16 mg g^−1^ DW on day 31.

**Figure 7:**
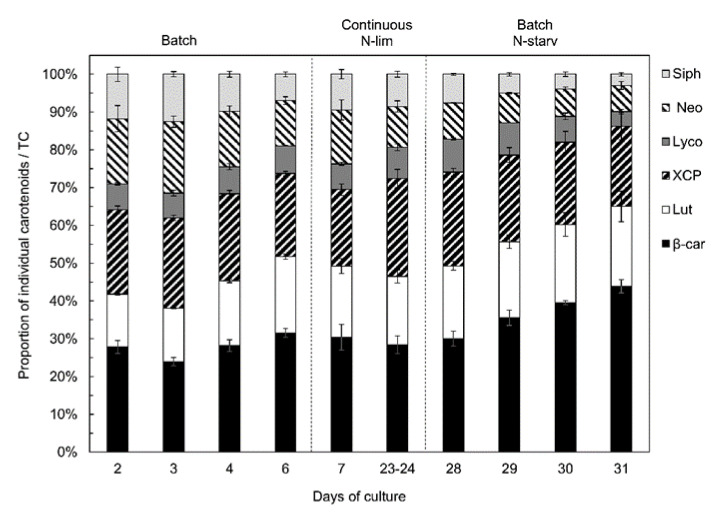
Variations of carotenoids proportion against total carotenoids in *Nephroselmis* sp. at different times of the culture. Siph, siphonaxanthin; Neo, neoxanthin (*trans* and *cis*); Lyco, lycopene; XCP, xanthophyll cycle pigments (violaxanthin + antheraxanthin + zeaxanthin); Lut, lutein; β-Car, β-carotene. Data are expressed as mean ± standard error (SE, *n* = 2).

If we consider the proportion of the different carotenoids against total carotenoids (Figure 7), the xanthophyll cycle pigments and lycopene proportion stayed stable at around 23 and 7%, respectively, throughout the experiment. However, the β-carotene and lutein proportions increased along with N-starvation, while the siphonaxanthin and neoxanthin proportions decreased sharply. At the end of the experiment, β-carotene and lutein reached up to 65% of the total carotenoids, while siphonaxanthin and neoxanthin represented almost 10%.

### 2.4. Correlation between Antioxidant Activity and Carotenoid Content

A correlation analysis was performed to assess a potential relationship between carotenoid content and peroxyl scavenging activity (Table 1). A good correlation was observed between total carotenoid content and antioxidant activity (Pearson’s correlation coefficient of 0.80). The adjusted R² indicates that the total carotenoid content explains 62% of the variability of the antioxidant activity measured in *Nephroselmis* sp. extracts. A closer look at individual carotenoids showed that they all contributed to the correlation with antioxidant activity, with different strengths—strong relationships were observed with siphonaxanthin, neoxanthin, and xanthophyll cycle pigments contents, while the relationships were weaker for lutein, lycopene, and β-carotene contents. A good correlation between chlorophyll and antioxidant activity was also found (correlation coefficient of 0.83 and R^2^ of 0.68), but this was attributed to the concomitant variation of chlorophyll with siphonaxanthin and neoxanthin.

### 2.5. Biomass and Carotenoid Productivities

Biomass and carotenoid productivities were calculated during the first batch culture period (day 2 to 6) and at steady state for the continuous culture. The highest biomass productivities were obtained on day 3 (120.8 ± 1.5 mg L^−1^ day^−1^) and in continuous mode at steady state at 0.3 days^−1^ of renewal (110.6 ± 5.7 mg L^−1^ day^−1^). The highest productivities for all individual pigments and total carotenoids were also measured on day 3 (siphonaxanthin, 0.77 ± 0.05 mg L^−1^ day^−1^; neoxanthin, 1.16 ± 0.08 mg L^−1^ day^−1^; xanthophyll cycle pigments, 1.47 ± 0.06 mg L^−1^ day^−1^; lutein, 0.87 ± 0.00 mg L^−1^ day^−1^; lycopene, 0.40 ± 0.04 mg L^−1^ day^−1^; β-carotene, 1.47± 0.08 mg L^−1^ day^−1^; total carotenoids, 6.13 ± 0.24 mg L^−1^ day^−1^).

## 3. Discussion

Nitrogen availability had a significant influence on the growth and elemental composition of *Nephroselmis* sp. After the first nitrate starvation (during the first batch period), the cell concentration of *Nephroselmi*s sp. still increased for one day (Figure 1), as reported in earlier studies [47,48]. The sustained growth during the early stage of N-starvation is explained by a redistribution of the endogenous pool of nitrogen (i.e., amino acids, proteins, chlorophyll, free nitrate) for synthesis of the nitrogen compounds that are essential to maintain cell division and survival [48,49,50]. However, a prolonged starvation period results in growth inhibition and ultimately cell death if nitrogen is not resupplied, as observed during the second batch period at the end of the experiment. Although growth was inhibited with N-starvation during the first batch culture, biomass production of *Nephroselmis* sp. still increased (Figure 3), which is probably linked to the increase of carbon cellular content (Figure 2). The C/N ratio under N-replete conditions (8.3 ± 0.1) exceeds Redfield ratio, which is assumed to be around 6.6 [51]. However, Geider and La Roche [52] showed that the C/N ratio of N-replete microalgae culture would rather be between 6.8 and 8.7 and would increase under N-limited conditions. In batch culture, cells adjust their metabolism to acclimate to N-starvation, but steady state cannot be reached if stress conditions persist, while in continuous culture, even if nitrogen limits growth, cells are able to acclimate to the low nitrogen concentration and steady state is reached. Thus, the physiological states of N-starved and N-limited cells are different, which is reflected by the elemental compositions [53,54]. The C/N ratio of *Nephroselmis* sp. increases up to 19.2 under N-starved conditions, while it stabilized at 14 in N-limited conditions. The increase of the C/N ratio in N-starved cells was driven by both a decrease of the nitrogen cellular content by 32% and an increase of carbon cellular content by 38%, while under N-limited conditions at steady state the carbon cellular content did not increase and nitrogen cellular content was higher than under N-starved condition, resulting in a lower C/N ratio. The increase of the carbon cell content is a common response of microalgae to N-starvation. Photosynthetic carbon fixation usually decreases, which generates an excess of carbon stored in nitrogen-free compounds, mostly neutral lipids and carbohydrates [55,56,57,58]. At steady state in continuous culture, carbon is used for cell growth and does not accumulate in the cells [56,59].

As for the growth and elemental composition, nitrogen variation also induced adjustments of the pigment contents and compositions for *Nephroselmis* sp. Indeed, during the first batch period, chlorophylls and total carotenoid contents increased along with the exponential cell growth and started to decrease once nitrogen was depleted in the culture medium. Both of these increased again when nitrate was resupplied (day 7) (Figure 5a,c). Nitrogen availability is known to impact the photosynthetic apparatus; in microalgae, N-limitation induces a reduction of protein synthesis, resulting in a preferential loss of chloroplastic proteins, and thus PSs proteins [47,60,61,62]. As a consequence, chlorophyll and carotenoids that are associated with the PSs decrease as well, which is consistent with the results observed for *Nephroselmis* sp. However, as a N-rich compound, chlorophylls of *Nephroselmis* sp. were more impacted by nitrogen availability, leading to an increase of the TC/Chl ratio under N-deprivation (Figure 5d). This is consistent with previous reports on other green microalgae [29,32,47,63].

High light intensity and N-starvation are known to have cumulative effects on pigment content. Under N-starvation, chlorophyll and primary carotenoids usually decrease further with high light intensity, while secondary carotenoid production is stimulated, such as for β-carotene in *Dunaliella* spp. [32,64,65]. In our experiment, nitrogen availability was the main factor that drove pigment variation. A shift of the light intensity from 600 to 1100 µmol m^−2^ s^−1^ prior to steady-state measurements (day 8) was implemented to avoid light limitation during continuous culture. The pigment contents of *Nephroselmi*s sp. were not significantly impacted, since their values were similar at day 6 and day 28 under identical nitrogen availability and culture method. N-limitation and an important self-shading effect owing to the high biomass that reduces the impact of light availability might explain this absence of difference in pigment contents.

*Nephroselmis* sp., similarly to the majority of microalgae species, reacted to nitrogen starvation or limitation by reducing its photosynthetic activity. As a result, the pool of photosynthetic pigments decreased. Siphonoxanthin, neoxanthin, lycopene, and xanthophyll cycle pigments followed similar variations as the chlorophyll contents in response to nitrogen availability, owing to their implication in the PSs.

The biological role of siphonaxanthin was not clearly described in green microalgae, but its functions were described in previous studies on siphonous green algae. This ketocarotenoid, firstly described by Yokohama [66], acted as an accessory light-harvesting pigment, absorbing blue-green light [67,68,69]. Chen et al. [68] and Wang et al. [69] showed in *Bryopsis corticulans* that siphonaxanthin was associated with PSI and chlorophylls *a* and *b* in a siphonaxanthin–chlorophyll ab–protein complex, and with the light-harvesting complex (LHC) of PSII with chlorophylls *a* and *b*, neoxanthin, and siphonaxanthin esters. The light harvesting complexes with siphonaxanthin are ancestral LHCs that evolved by replacing siphonaxanthin with lutein and xanthophyll cycle pigments in higher plants to adapt to high light environments [70]. The strong correlation between siphonaxanthin and neoxanthin contents and chlorophyll contents observed for *Nephroselmis* sp. (R² = 0.98, *p* < 0.001 for siphonaxanthin; R² = 0.97, *p* < 0.001 for neoxanthin) suggests that the LHCs of *Nephroselmis* sp. might be similar to LHCs of *Bryopsis corticulans*. However, unlike *Bryopsis, Nephroselmis sp*. also contains xanthophyll cycle pigments and a high concentration of lutein; this species might, thus, be at the intermediate stage between primitive chlorophytes with siphonaxanthin-type LHCs and higher plants [38,70].

Yoshii et al. [38] classified species of *Nephroselmis* into five distinct types according to their carotenoid compositions, especially siphonaxanthin and its derivatives. Indeed, siphonaxanthin derivatives were found among all the *Nephroselmis* species studied by Yoshii et al. [38], such as siphonaxanthin esters or methoxy siphonaxanthin. However, we did not find any siphonaxanthin derivatives for any culture conditions in our strain (present study and Coulombier et al. [37]), and we could not classify it according to Yoshii et al.’s classification [38]. Thus, we suggest that this species could belong to a sixth type that contains only siphonaxanthin and no siphonaxanthin derivatives.

As with the siphonaxanthin and neoxanthin contents, the xanthophyll cycle pigments and lycopene content were higher in *Nephroselmis* sp. under N-replete conditions (Figure 6). Information about lycopene is scarce, since it is usually undetected in microalgae [17], but it represented around 7% of total carotenoids in *Nephroselmis* sp. (Figure 7). Lycopene is a precursor of α and β-carotenes, but several studies have demonstrated its antioxidant properties as one of the most efficient quenchers of singlet oxygen [71,72,73,74,75], suggesting a photoprotective function. On the contrary, the response of the xanthophyll cycle pigment content to nitrogen availability has been well studied, and our results are consistent with previous reports [29,63,64,76].

Unlike the other carotenoids (neoxanthin, siphonaxanthin, lycopene, and the xanthophyll cycle pigments), β-carotene and lutein contents did not follow the same variation as the chlorophyll content. The β-carotene and lutein are known to act as primary carotenoids with photoprotective functions [17,77,78]. Therefore, for most chlorophytes species, N-starvation induces a decrease of lutein [32,63,79,80,81] and β-carotene contents [31,82], along with chlorophyll. Unexpected results were obtained for *Nephroselmis* sp., since the lutein content remained stable, regardless of the nitrogen availability, suggesting that this pigment is not associated with the PSs (Figure 6). Some chlorophyte species (i.e., *Dunaliella salina* or *Parietochloris incisa*) are known to have the ability to accumulate β-carotene in extra-thylakoid lipid droplets when subjected to N-starvation, along with other stressors such as high light intensity [17,32,83]. This could explain the stability of the β-carotene content in *Nephroselmis* sp. under stressful conditions; however, to the best of our knowledge there is no report of extra-thylakoid lutein accumulation. Additional investigations are necessary to clarify the localization and the physiological role of lutein in *Nephroselmis* sp.

As for growth and pigment content, nitrogen availability strongly impacted the antioxidant activity (peroxyl radical scavenging activity) of the *Nephroslemis* sp. extract. Indeed, the *Nephroselmis* sp. extract was twice as active under N-replete conditions than under N-limited or starved conditions (Figure 4). Those results are in agreement with the few studies that have evaluated the effects of N-limitation on antioxidant activities [25,29,35,36]. Çakmak et al. [25] and Aremu et al. [35,36] showed the negative impacts of N-starvation on the antioxidant activity of *Chlamydomonas reinhardtii* and *Chlorella* strains, respectively. Goiris et al. [29] found an overall antioxidant activity that was 3 to 10 times higher in N-replete cultures than in the N-starved cultures of 3 microalgae species (*Chlorella vulgaris*, *Tetraselmis suecica*, and *Phaeodactylum tricornutum*). As phenolic and carotenoid contents showed similar responses to nitrogen levels, this suggests that these compounds were the main contributors to the antioxidant activity. Our results showed a clear correlation between the peroxyl radical scavenging activity of the *Nephroselmis* sp. extract and the total carotenoid content (Table 1), in agreement with our previous report [37]. Peroxyl radical scavenging is essential to protect cellular membranes against oxidative damage, since peroxyl radicals can start lipid peroxidation chain reactions [14]. All carotenoids seem to contribute to the peroxyl scavenging activity of *Nephroselmis* sp. extract, which is consistent with several reports that have shown the peroxyl scavenging activity of a variety of carotenoids [84,85,86]. However, the strongest correlation was measured for siphonaxanthin and neoxanthin, suggesting that these two carotenoids had a higher activity, or that at least one of them did, since they followed the same variations. The efficiency of each carotenoid toward peroxyl radicals is known to be related to their specific structure, such as the number of conjugated double bonds, the type of terminal group, the presence of oxygen substituents, or the *cis-trans* isomer configuration [84,85]. Despite some conflicting results due to the use of different protocols, all studies agreed that ketocarotenoids are among the most efficient carotenoids in terms of action against peroxyl radicals, while hydroxycarotenoids and β-ionone carotenes are less active [84,85,86]. In addition, Dambeck and Sandmann [39] have shown that siphonaxanthin exerts an efficient effect against radical formation and lipid peroxidation, and our previous results on *Nephroselmis* sp. showed its high peroxyl radical scavenging activity, although neoxanthin was not detected in the methanol or dichloromethane extracts [37]. These results suggest a higher implication of siphonaxanthin than neoxanthin in the peroxyl scavenging activity of *Nephroselmis* sp. extract, but this needs to be confirmed by measures of peroxyl radical scavenging activity for purified siphonaxanthin and neoxanthin. The other carotenoids appeared to be less efficient in scavenging peroxyl radicals; however, they are known to be implied in the cell’s protection against oxidative stress by other mechanisms. Xanthophyll cycle pigments are able to quench singlet-excited chlorophyll and dissipate the excess of energy via heat , lutein and β-carotene can deactivate triplet-excited chlorophyll to prevent formation of ROS, and all carotenoids are able to quench ROS directly (especially for singlet oxygen), with different efficiencies depending on the number of conjugated double bonds [17,78,87].

In order to estimate the best nitrogen supply conditions for carotenoid production, the biomass and carotenoid productivities in *Nephroselmis* sp. were determined (Table 2). The highest biomass productivities were obtained in the late exponential phase of the first batch culture (day 3) (120.8 ± 1.5 mg L^−1^ day^−1^) and in continuous culture at steady state (110.6 ± 5.7 mg L^−1^ day^−1^). However, as the carotenoid content was 62.5% higher in the first batch culture than at steady state, the total carotenoid productivity increased by 80% between the two conditions. The best productivity was also achieved on day 3 for all individual carotenoids. Thus, cultivation under N-replete conditions is essential to obtain the highest carotenoid productivity and radical peroxyl scavenging activity in *Nephroselmis* sp.

The high total carotenoid contents of up to 5% of the cell’s dry weight with pigments of interest, such as lutein and siphonaxanthin, and the high antioxidant activity make *Nephroselmis* sp. a species of interest. Indeed, the peroxyl radical scavenging activity of *Nephroselmis* sp., at between 63.6 and 154.9 µmol TE g^−1^ DW, was among the highest values reported previously with ORAC assays of microalgae crude extracts. Banskota et al. [88] found ORAC values of between 6.69 and 52.98 µmol TE g^−1^ DW in methanol extracts of nine microalgae species (*Nannochloropsis granulata*, *Phaeodactylum tricornutum*, *Tetraselmis chui*, *Botryococcus braunii*, *Chlorella sorokiniana*, *Neochloris oleoabundans*, *Porphyridium aerugineum*, *Scenedesmus obliquus*, and *Scenedesmus* sp.). An antiperoxyl radical activity of 61.53 µmol TE g^−1^ DW was reported in 50% water acetone extract of *Phormidium autumnale* [89], while ORAC values of 31.21 and 12.2 µmol TE g^−1^ DW were measured in 50% water ethanolic extracts of *Chlorella vulgaris* and *Spirulina platensis*, respectively [90]. Ahmed et al. [91] found values in the same range as *Nephroselmis* sp. (45 to 288 µmol TE g^−1^ DW) in hexane extracts of eleven microalgae species (*Dunaliella salina*, *Isochrysis galbana*, *Nannochloropsis sp*., *Pavlova lutheri*, *Pavlova salina*, *Chaetoceros muelleri*, *Tetraselmis chui*, *Tetraselmis suecica*, *Tetraselmis sp*., *Phaeodactylum tricornutum*, and *Dunaliella tertiolecta*); however, peroxyl radical scavenging activity was higher in water (60 to 350 µmol TE g^−1^ DW) and ethyl acetate extracts (169 to 577 µmol TE g^−1^ DW). Furthermore, the lutein content of *Nephroselmis* sp. at up to 7.97 ± 0.84 mg g^−1^ DW was within the upper range values reported for lutein-producing strains, ranging between 0.5 and 9.6 mg g^−1^ DW [79,80,81,92,93,94]. However, further work on culture conditions is necessary to improve the biomass productivity, and thus the lutein productivity, in order to reach the levels reported in previous studies (up to 4.9 mg L^−1^ day^−1^) [79,81,95,96,97]. In addition to antioxidant activity, siphonaxanthin has interesting bioactive properties, including induction of apoptosis of human leukemia cells [45], along with antiangiogenic [40], antiobesity [41,42,43], and anti-inflammatory effects [44]. The siphonaxanthin contents in *Nephroselmis* sp. were 6–20% higher than contents reported in green algae [98], indicating *Nephroselmis* sp. as a species of interest for siphonaxanthin production.

## 4. Materials and Methods

### 4.1. Strain

*Nephroselmis sp.* N3C46 (Prasinophytina, Chlorophyta, Supplementary Materials Figure S4) was isolated in tropical seawater in a lagoon in New Caledonia (authorization no. 26960, delivered by the South Province of New Caledonia) [37]. Inocula were grown for 7 days in a 1-L air-bubbled Erlenmeyer flask in 0.2 µm filtered sterilized seawater enriched with 1 mL L^−1^ of Walne’s medium [99]. The continuous light intensity was set to 600 µmol m^−2^ s^−1^ using a Li-cor quantum meter (LI-250A US-SQS/L with a spherical probe) at a temperature of 26.5 °C.

### 4.2. Nephroselmis sp. Molecular Identification

Microalgal DNA was extracted using an optimized phenol-chloroform method [100]. Briefly, cells were centrifuged (2000× *g*, 10 min, 4 °C). The pellet was washed twice with TE-NaCl buffer (Tris-HCl 0.1 M, EDTA 0.05 M, NaCl 0.1 M, pH 8.0). After overnight incubation with TE-NaCl, the pellet was pretreated with buffer lysis (1% SDS, 1% Sarkozyl, 400 µg mL^−1^ Proteinase K) for 2 h at 40 °C. The extract was purified with equal volumes of phenol/chloroforme/isoamyl alcohol mixture (PCA, 25:24:1) and centrifuged (8600 g, 20 min, 4 °C). The upper aqueous layer was further purified with an equal volume of chloroform and centrifuged (8600× *g*, 20 min, 4 °C). The aqueous phase containing DNA was then pretreated by RNase (8 µg mL^−1^, 1 h, 60 °C). After a second step of PCA extraction, DNA was precipitated and washed with isopropanol and 70% ethanol, respectively. Then, the DNA pellet was solubilized in 100 µL of DNase-free water. The concentration and quality of the extracted genomic DNA were measured using a NanoDrop Spectrophotometer (Thermo Scientific, Wilmington, DE, USA). The amplification of the microalgal 18S rRNA was done using a universal primer pair of 18S-F (5′-ACCTGGTTGATCCTGCCAGT-3′) and 18S-R (5′-TCCTTCTGCAGGTTCACCTAC-3′). The PCR reaction was performed at a final volume of 50 μL, which included Green GoTaq^®^ Reaction Buffer (Promega, Madison, WI, USA), MgCl_2_ (1.5 mM); dNTPs (0.2 mM), GoTaq G2 (0.05 µg µL^−1^, Promega, Madison, WI, USA), primer forward (1 µM); primer reverse (1 µM), and extracted DNA (0.01 ng µL^−1^). The PCR amplification was then performed in a thermocycler (Mycycler, BioRad, Hercules, CA, USA). The PCR product was then examined in 1% agarose gel and subsequently cloned (band at 1800 bp) with a TOPO TA cloning kit (Invitrogen, Carlsbad, CA, USA ref: K457501). The clones were sequenced on Sanger ABI at Eurofins Genomics (Paris, France). The DNA sequence was then compared with Basic Local Alignment Search Tool (BLAST; blast.ncbi.nlm.nih.gov [101]) for taxonomic identification. In addition, the *Nephroselmis* 18S rRNA sequence was placed with a representative selection of *Nephroselmis* spp. sequences taken from GenBank (similarly to [102,103] with diatoms). The microalgal 18SrRNA sequence was deposited in GenBank (GenBank accession number: MT833289).

### 4.3. Culture Conditions and Experimental Protocol

Experiments were carried out in two 10 L photobioreactors (PBRs) made of transparent polymethylmethacrylate. The temperature was kept constant at 26.5 °C ± 0.3 and the pH was regulated at 7.75 ± 0.04 by automated CO_2_ addition. Light was provided on one side of the PBRs using seven adjustable fluorescent light tubes (OSRAM cool 109 daylight HO24W/965). Continuous light was set at 600 µmol m^−2^ s^−1^ by measuring the light intensity inside the PBRs. High light irradiance was used on the basis of previous results [37] that reported a better antioxidant activity of *Nephroselmis sp*. with high light intensity. A Rushton turbine at 60 rpm and 0.2 µm filtered air bubbling were used to homogenize the culture. Before inoculation, PBRs were sterilized for 20 min with a 5‰ peroxyacetic acid solution and rinsed twice with 0.2 µm filtered seawater. PBRs were inoculated with 1 L of inoculum and filled up to 9.5 L with 0.2 µm filtered seawater enriched with 1 mL L^−1^ of Walne’s medium at 1.18 mM-N to reach an initial concentration of 7 × 10^5^ cell mL^−1^.

The experiment was separated in 3 successive stages with various nitrogen conditions (Figure 1):

(1) A first batch culture period (day 0 to day 6) was applied to study the effects of N-replete to N-starvation conditions. At the end of this period, and owing to sample collections during this first batch culture, PBRs volumes were restored to 9.5 L (day 6) by adding filtered sea water (0.2 µm) enriched with 1 mL L^−1^ of Walne’s medium at 1.18 mM-N to ensure that the temperature and pH probes remained submerged;
(2) The PBRs were then switched to a continuous mode of culture in chemostat to study the effects of N-limitation (day 7 to day 27). During this period, a dilution rate of 0.3 day^−1^ was applied. The culture medium was composed of filtered seawater (0.2 µm) enriched in 1 mL L^−1^ of Walne’s medium at 1.18 mM-N [99]. The light intensity was increased to 1100 µmol m^−2^ s^−1^ on day 8 to ensure that there was no light limitation owing to the very high cellular concentration (>25 × 10^6^ cell mL^−1^). Continuous culture was maintained until analyses at steady state (i.e., at least 3 days with less than 10% variation of cellular concentration and absorbance);
(3) Finally, the PBRs were switched back to a second batch culture period (day 28 to day 31) in N-starvation conditions until beginning of senescence.

### 4.4. Cell Growth Measurements

Growth was followed using two methods and performed daily. Cells were counted using a Malassez hemocytometer under an optical microscope and light absorbance was measured at 680 and 800 nm for chlorophyll *a* absorption and non-pigmented cell compounds absorption, respectively (see light absorbance at 680 nm over time and correlation analysis between cell concentration and light absorbance at 680 nm in Supplementary Materials Figures S5 and S6).

### 4.5. Particulate Organic Carbon and Nitrogen and Residual Nitrate and Phosphate

Carbon and nitrogen cell contents and residual nitrate and phosphate in the medium were determined daily. For C and N analysis, samples of 2 to 20 mL of culture were filtered through pre-combusted glass filters (1.2 μm, Whatman GF/C), then filters were dried at 70 °C for 24 h and kept at −20 °C until analysis by a CHN (Carbon Hydrogen Nitrogen) elemental analyzer (SERCON Integra 2). For residual nitrate and phosphate, 10 mL of culture medium was filtered through a 0.2 µm filter and the filtrate was kept at −20 °C until analyses using continuous flow auto analyzer (AA3 Seal Analytical)

### 4.6. Measurement of Antioxidant Activity and Pigments Analysis

#### 4.6.1. Sampling

In each PBR, samples of 1 L of culture were collected at different steps of the culture to determine the dry weight, antioxidant activity, and pigment composition. During the first batch culture, two samples were collected during the exponential growth phase (days 2 and 3) and two sample were collected during the stationary phase (days 4 and 6). During continuous culture, one sample was collected after refilling the PBRs (day 7) and two samples were collected and pooled at steady state (days 23 and 24). During the second batch culture, samples were collected daily until senescence (days 28, 29, 30, and 31).

For all samples, microalgae were harvested by centrifugation (4500× *g*, 10 min, 4 °C), freeze-dried, and kept at −80 °C until extraction. Dry weight (DW) was determined by weighing the total amount of harvested biomass that was freeze-dried.

#### 4.6.2. Antioxidant Activity

Extracts for antioxidant activity determination were obtained by maceration in ethanol. In the dark and at room temperature, 50 mg of freeze dried biomass was ground using a pestle and mortar and then suspended in 5 mL of ethanol. The solution was placed at −20 °C in a closed container for 30 min in darkness to limit oxidation, then the extract was centrifuged (4500× *g*, 5 min, 4 °C). The supernatant was conserved and the pellet was resuspended in 2 mL of ethanol and centrifuged again. The procedure was repeated until the pellet remained colorless (two to three times). The supernatants of each extract were pooled, dried under a stream of nitrogen, and stored under nitrogen atmosphere at −80 °C until analysis. On the basis of a previous study, the oxygen radical absorbance capacity (ORAC) assay was selected to determine the antioxidant activity [37]. The ORAC assay measures the scavenging capacity of an antioxidant against peroxyl radicals by hydrogen atom transfer. Thermal decomposition of 2,2′-azobis-(2-amidinopropane) dihydrochloride (AAPH) leads to the formation of peroxyl radicals, which react with fluorescein (fluorescent probe). This causes a fluorescence loss that is measured over time [104]. A method adapted from Watanabe et al. [105] was applied on *Nephroselmis sp.* extracts [37]. Extracts and Trolox (standard) were first diluted in DMSO (5.7 mg mL^−1^). Then, 10% of the extracts or Trolox solutions were mixed with 90% (*v/v*) of a diluent solution made of 7% (*w/v*) of randomly methylated β-cyclodextrin (RMCD) in 50% (*v/v*) acetone aqueous solution. Then, each extract and Trolox were diluted again with DMSO/diluent solution (10:90 *v/v*) to obtain 3 different concentrations (50, 25, and 12.5 µg mL^−1^) and a Trolox concentration range of 0.5 to 10 µg mL^−1^ to make a calibration curve. In a black 96-well plate, 35 µL of each sample was placed in the wells. A blank was made with the same volume of DMSO/diluent solution (10:90 *v/v*). Fluorescein (115 µL, 77.5 nM) was added to the wells and the plate was incubated at 37 °C for 10 min with 20 rpm agitation. Then, 50 µL of AAPH (82.4 mM) was added and the fluorescence was measured for 300 min every 2 min at an excitation wavelength of 485 nm and emission wavelength of 528 nm.

For each sample and the blank, the area under the curve (AUC) was calculated with the formula from Huang et al. [106]:

(1) $$\mathrm{AUC}=0.5+\frac{f_{1}}{f_{0}}+\cdots \frac{f_{i}}{f_{0}}+\cdots +\frac{f_{298}}{f_{0}}+0.5\;\left(\frac{f_{300}}{f_{0}}\right)$$

where *f_o_* is the initial fluorescence and *f_i_* is the fluorescence at time *i*. The net AUC was obtained by subtracting the AUC of the blank from the AUC of the sample. The antioxidant activity values of the extracts were computed by linear regression on a Trolox calibration curve obtained by plotting the Trolox concentration vs. net AUC. The results are expressed as ORAC values in µmol Trolox equivalent g^−1^ of dried weight biomass (µmol TE g^−1^ DW).

#### 4.6.3. Pigments Analysis

For pigment analysis, extraction was performed on 40 mg of fresh dried biomass that was previously ground using a mortar and pestle to obtain a fine powder, then suspended in 4 mL of absolute ethanol. The mixture was homogenized using a vortex, then 0.5 mL was immediately sampled and mixed with 100 mg of 150–400 µm glass beads in a mixer miller (Retsch MM-400) for 10 min at a frequency of 30 Hz. After centrifugation (16,000× *g*, 5 min, 6 °C), the supernatant was conserved and the pellet was resuspended using a vortex with 500 µL of ethanol, then centrifuged again. This procedure was repeated twice until the pellet remained colorless. The three supernatants were then pooled and filtered on a 0.2 µm PTFE (polytetrafluoroethylene) filter prior to HPLC analysis. The samples were analyzed following the method of Van Heukelem and Thomas [107] by HPLC-UV-DAD (High-Performance Liquid Chromatography-Ultra Violet-Diode Array Detector) (Agilent Technologies series 1200 HPLC-UV-DAD) using an Eclipse XDB-C8 reverse-phase column (150 × 4.6 mm, 3.5 µm particle size, Agilent). Pigment identification was done using a spectral library published by Serive et al. [108]. Quantification was carried out using external calibration against pigment standards (lutein, neoxanthin, violaxanthin, antheraxanthin, zeaxanthin, β-carotene, lycopene, fucoxanthine, chlorophylls *a* and *b*, purchased from DHI, Denmark). Quantification of siphonaxanthin was done according to fucoxanthin standard as recommended by Roy et al. [109]. Moreover, the type of siphonaxanthin was identified by UV-Vis spectrum in a HPLC system and mass spectroscopy analysis in our previous study [37].

### 4.7. Statistical Analysis

Following an examination of the homogeneity of variance and normal distribution (Kolmogorov–Smirnov test), a one-way ANOVA was performed and differences were considered significant at *p* (α = 0.05) < 0.05. A Fisher’s least significant difference (LSD) test was then applied to determine which experimental conditions were significantly different. To study the correlation between carotenoid content and antioxidant activity, a Pearson’s correlation test was used. Statistical analyses were carried out using Statgraphics Centurion XV.I (StatPoint Technologies, Inc., Warrenton, VI, USA).

## 5. Conclusions

Pigment analysis of *Nephroselmis* sp. revealed a specific composition of the photosynthetic system at the crossroads of primitive green algae and higher plants. The correlation of siphonaxanthin and neoxanthin with chlorophylls suggests the presence of siphonaxanthin-type LHCs, as in primitive green algae. However, the results showed the presence of xanthophyll cycle pigments and lycopene, which are also implied in the PSs, as well as the presence of a large amount of lutein, as in most advanced chlorophytes. Surprisingly, lutein did not seem to be associated with the PSs.

Our results with *Nephroselmis* sp. highlight that the N-replete condition leads to high peroxyl radical scavenging activity, primary carotenoid contents, and productivities with *Nephroselmis* sp. Indeed, a 3-fold increase was found for the primary carotenoid content and productivity, while a 2.4-fold increase for antioxidant activity under N-replete conditions. Lutein and β-carotene contents were not influenced by nitrogen availability, but their productivities, which followed biomass productivity, were also higher under N-replete conditions. In addition, the results showed high peroxyl radical scavenging capacity that was linked to the carotenoid content of *Nephroslemis* sp., in particular to the siphonaxanthin and neoxanthin contents. This high antioxidant activity owing to the high carotenoid content makes *Nephroselmis* sp. a species of interest for carotenoid production as natural antioxidants, especially lutein and siphonaxanthin. The impacts of other culture parameters such as pH or temperature have not been investigated yet, but it would be interesting to study their effects to further improve the carotenoid content and productivity of *Nephroselmis* sp.

## Figures and Tables

**Table 1:** Pearson’s correlation test between carotenoid content (mg g^−1^ DW) and antioxidant activity measured with ORAC assay (µg Trolox equivalent mg^−1^ DW) in *Nephroselmis* sp. Siph, siphonaxanthin; Neo, neoxanthin (*trans* and *cis*); XCP, xanthophyll cycle pigments (violaxanthin + antheraxanthin + zeaxanthin); Lut, lutein; Lyco, lycopene; β-Car, β-carotene.

	Antioxidant Activity	
	Correlation Coefficient	Adjusted R²
Siph	0.82 **	0.66
Neo	0.82 **	0.66
XCP	0.75 **	0.55
Lut	0.51 *	0.22
Lyco	0.65 **	0.40
β-car	0.60 *	0.33
Total carotenoids	0.80 **	0.62

Note: * *p* < 0.05; ** *p* < 0.001; *n* = 22.

**Table 2:** Biomass and carotenoid productivity (mg L^−1^ day^−1^) of *Nephroselmis* sp. during first batch culture and at steady state of continuous culture. Siph, siphonaxanthin; Neo, neoxanthin (*trans* and *cis*); XCP, Xanthophyll Cycle Pigments (violaxanthin + antheraxanthin + zeaxanthin); Lut, lutein; Lyco, lycopene; β-Car, β-carotene; TC, total carotenoids. Data are expressed as mean ± standard error (SE, *n* = 2). Different letters indicate statistically significant differences (*p* < 0.05).

		Biomass	Siph	Neo	XCP	Lut	Lyco	β-car	TC
Batch	Day 2	89.7 ± 0.7 ^c^	0.45 ± 0.08 ^b^	0.66 ± 0.10 ^b^	0.85 ± 0.05 ^bc^	0.53 ± 0.01 ^c^	0.26 ± 0.01 ^b^	1.06 ± 0.07 ^bc^	3.82 ± 0.30 ^bc^
	Day 3	120.8 ± 1.5 ^a^	0.77 ± 0.05 ^a^	1.16 ± 0.08 ^a^	1.47 ± 0.06 ^a^	0.87 ± 0.00 ^a^	0.40 ± 0.04 ^a^	1.47 ± 0.08 ^a^	6.13 ± 0.24 ^a^
	Day 4	108.4 ± 1.1 ^b^	0.44 ± 0.04 ^b^	0.66 ± 0.05 ^b^	1.04 ± 0.05 ^b^	0.77 ± 0.03 ^ab^	0.32 ± 0.03 ^ab^	1.27 ± 0.08 ^ab^	4.50 ± 0.22 ^b^
	Day 6	95.5 ± 2.6 ^c^	0.22 ± 0.03 ^c^	0.39 ± 0.03 ^c^	0.71 ± 0.04 ^b^	0.65 ± 0.04 ^bc^	0.24 ± 0.01 ^b^	1.02 ± 0.07 ^bc^	3.22 ± 0.21 ^c^
Continuous at steady state (Days 23–24)		110.6 ± 5.7 ^ab^	0.29 ± 0.02 ^bc^	0.37 ± 0.03 ^c^	0.88 ± 0.09 ^bc^	0.61 ± 0.08 ^c^	0.27 ± 0.05 ^b^	0.98 ± 0.06 ^c^	3.40 ± 0.34 ^c^

## Supplementary Materials

The following are available online at https://www.mdpi.com/1660-3397/18/9/453/s1: Figure S1. Phylogenetic tree of *Nephroselmis* sp. N3C46 based on 18S rRNA sequences. Figure S2: Residual phosphate concentration (µM) in the medium over time of *Nephroselmis* sp. cultures in PBRs in batch and continuous modes. Black dots represent sample collection for antioxidant activity measurement and carotenoid analysis. Data are expressed as mean ± standard error (SE, *n* = 2). Figure S3. HPLC chromatogram at 450 nm of ethanol extract of *Nephroselmis* sp. Siph, siphonaxanthin; Neo, neoxanthin; Viola, violaxanthin; Anthe, antheraxanthin; Zea, zeaxanthin; Lut, lutein; Chl, chlorophyll; Lyco, lycopene; β-car, β-carotene; Car 54, unidentified carotenoid (see Serive et al. [103] for UV-*vis* spectrum of Car 54 in HPLC system). Figure S4. Microphotography of *Nephroselmis* sp. Figure S5. Absorbance at 680 nm and residual nitrate concentration (µM) over time of *Nephroselmis* sp. cultures in PBRs in batch and continuous modes. Black dots represent sample collection for antioxidant activity and carotenoid analysis. Data are expressed as mean ± standard error (SE, *n* = 2), Figure S6. Pearson’s correlation analysis between cell concentrations in cells/mL^−1^ and light absorbance at 680 nm. Table S1: Pigment composition of *Nephroselmis* sp. (mg g^−1^ DW) at different times of the culture. Siph, siphonaxanthin; Neo, neoxanthin (trans and cis); XCP, Xanthophyll Cycle Pigments (violaxanthin + antheraxanthin + zeaxanthin); Lut, lutein; Lyco, lycopene; β-Car, β-carotene; TC, total carotenoids; Chl a, chlorophyll a; Chl b, chlorophyll b. Data are expressed as mean ± standard error (SE, n = 2). Different letters indicate statistically significant differences (*p* < 0.05).
