# Supplementary material for: Effects of Nitrogen Availability on the Antioxidant Activity and Carotenoid Content of the Microalgae Nephroselmis sp
Source: Mar Drugs. 2020 Aug 29;18(9):453. doi: 10.3390/md18090453 (PMC7551860; doi:10.3390/md18090453)
Supplement: Supplementary file 1 [file marinedrugs-18-00453-s001.pdf]

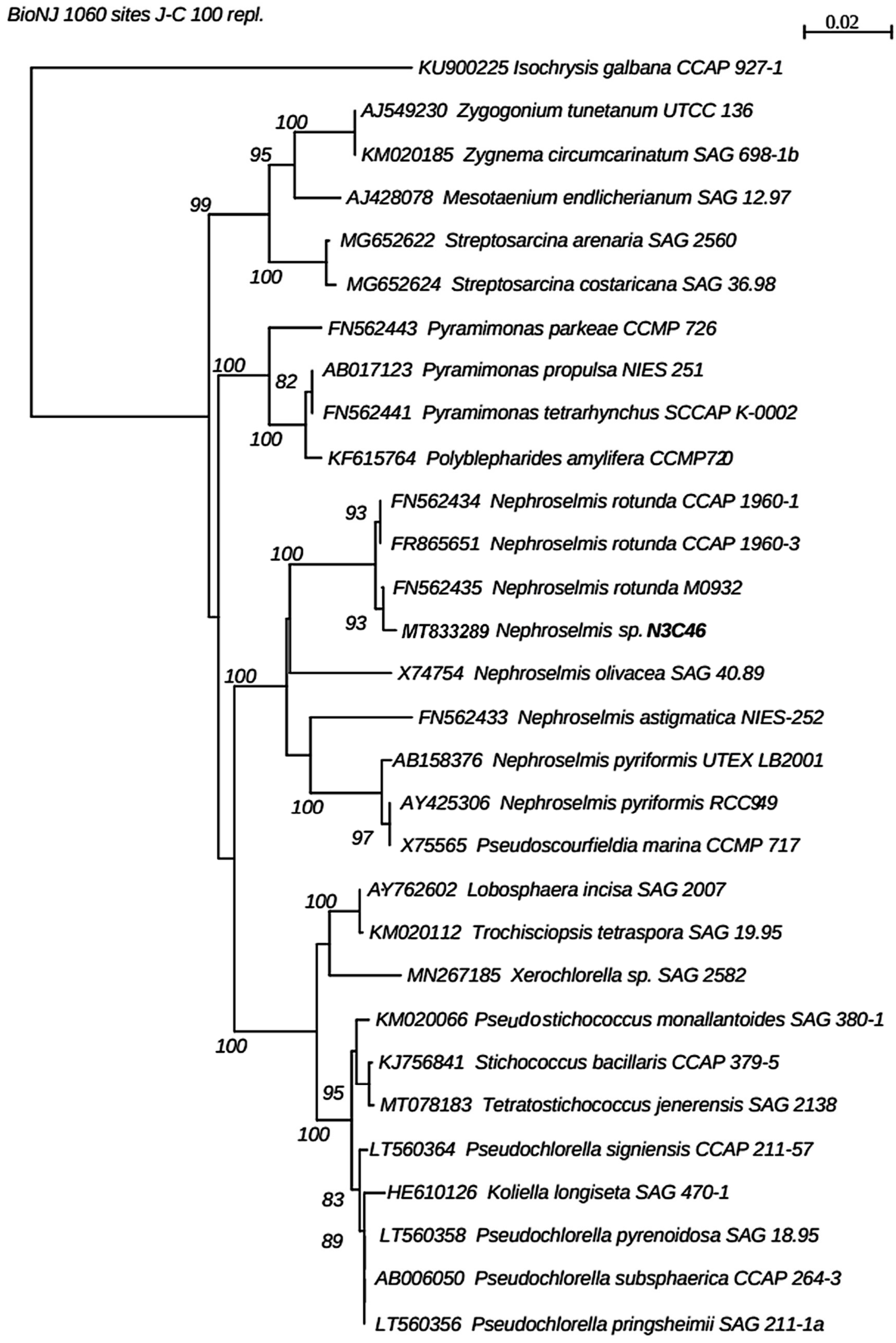

Figure S1. Phylogenetic tree of *Nephroselmis* sp. N3C46 based on 18S rRNA sequences

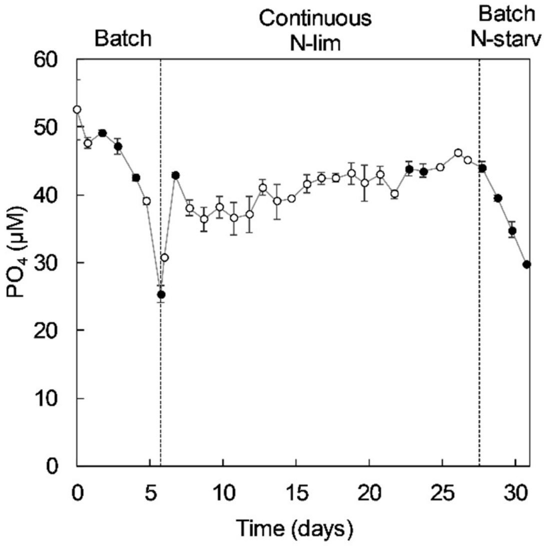

Figure S2. Residual phosphate concentration (µM) in the medium over time of *Nephroselmis* sp. cultures in PBRs in batch and continuous mode. Black dots represent sample collection for antioxidant activity measure and carotenoids analysis. Data are expressed as mean ± standard error (SE, n = 2).

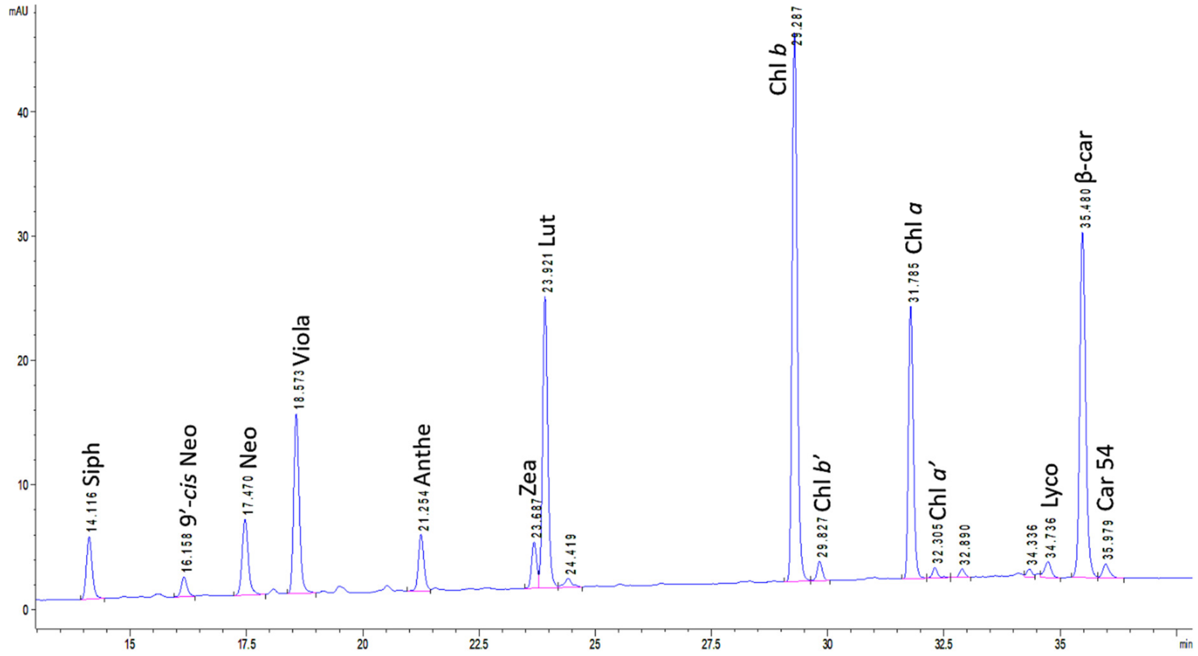

Figure S3. HPLC chromatogram at 450 nm of ethanol extract of *Nephroselmis* sp.. Siph, siphonaxanthin; Neo, neoxanthin; Viola, violaxanthin; Anthe, antheraxanthin; Zea, zeaxanthin; Lut, lutein; Chl, chlorophyll; Lyco, lycopene; β-car, β-carotene; Car 54, unidentified carotenoid (see Serive et al. [103] for UV-vis spectrum of Car 54 in HPLC system)

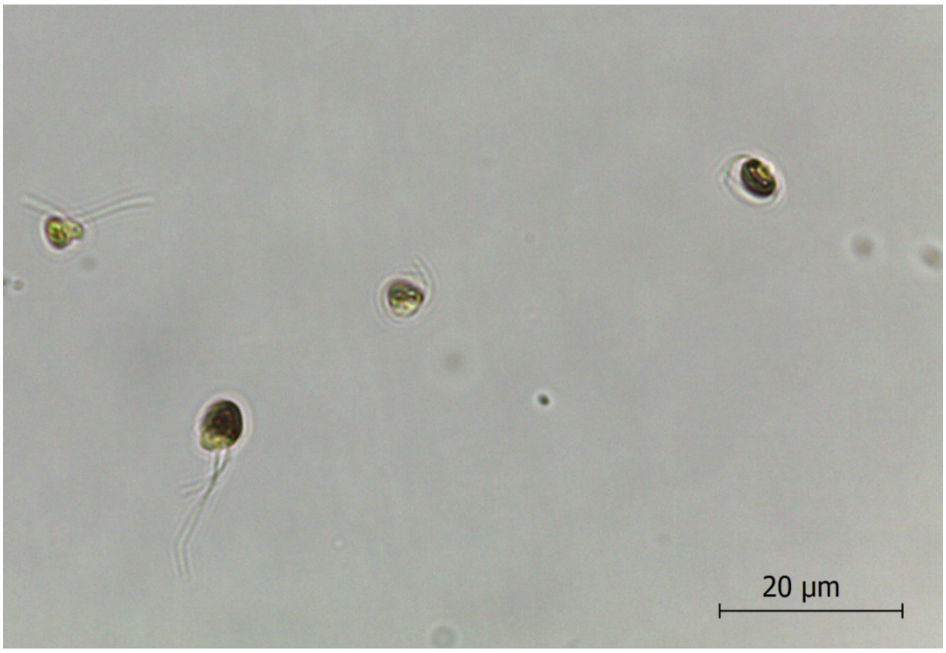

Figure S4. Microphotography of *Nephroselmis* sp.

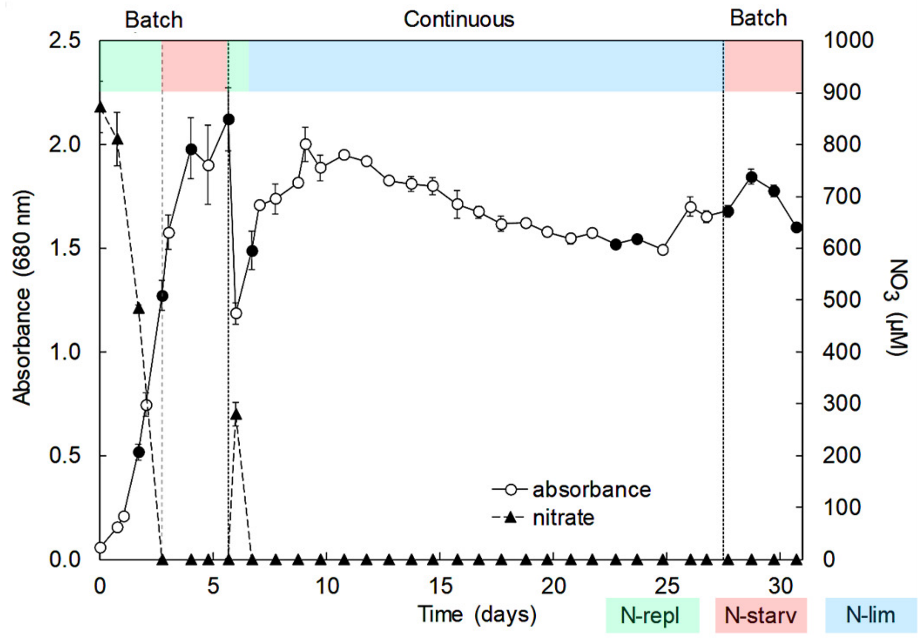

Figure S5. Absorbance at 680 nm and residual nitrate concentration (µM) over time of *Nephroselmis* sp. cultures in PBRs in batch and continuous mode. Black dots represent sample collection for antioxidant activity and carotenoids analysis. Data are expressed as mean ± standard error (SE, n = 2).

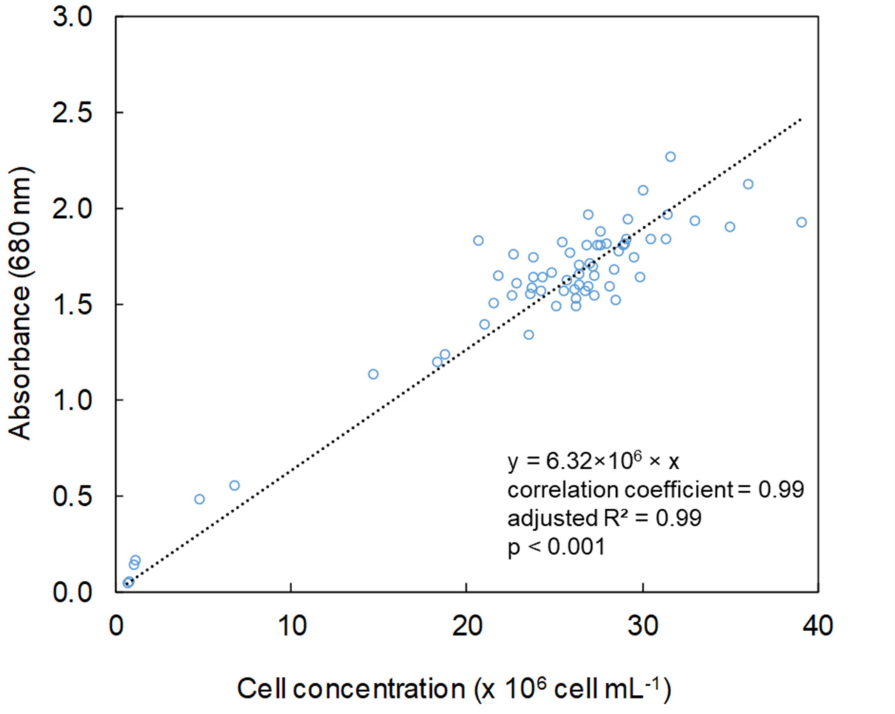

Figure S6. Pearson's correlation analysis between cell concentration in cell mL^{-1} and light absorbance at 680 nm

Table S1. Pigment composition of *Nephroselmis* sp. (mg g^{-1} DW) at different time of the culture. Siph, siphonaxanthin; Neo, neoxanthin (*trans* and *cis*); XCP, Xanthophyll Cycle Pigments (violaxanthin + antheraxanthin + zeaxanthin); Lut, lutein; Lyco, lycopene; β-Car, β-carotene; TC, total carotenoids; Chl *a*, chlorophyll *a*; Chl *b*, chlorophyll *b*. Data are expressed as mean ± standard error (SE, n = 2). Different letters indicate statistically significant differences ($p<0.05$).

			**Siph**	**Neo**	**XCP**	**Lut**	**Lyco**	**β-car**	**TC**	**Chl *a***	**Chl *b***
**Batch**		Day 2	5.00 ± 0.80^{b}	7.35 ± 1.47^{b}	9.50 ± 0.44^{b}	5.93 ± 0.10^{bc}	2.93 ± 0.12^{abc}	11.84 ± 0.73^{ab}	42.55 ± 2.99^{b}	73.75 ± 11.24^{b}	79.48 ± 6.40^{b}
		Day 3	6.36 ± 0.33^{a}	9.58 ± 0.75^{a}	12.14 ± 0.35^{a}	7.16 ± 0.06^{ab}	3.32 ± 0.37^{a}	12.14 ± 0.55^{ab}	50.70 ± 1.34^{a}	110.15 ± 3.79^{a}	102.97 ± 0.51^{a}
		Day 4	4.07 ± 0.32^{b}	6.08 ± 0.61^{b}	9.57 ± 0.36^{b}	7.13 ± 0.21^{ab}	2.99 ± 0.34^{ab}	11.71 ± 0.65^{ab}	41.55 ± 1.64^{b}	65.57 ± 2.81^{bc}	64.21 ± 3.30^{c}
		Day 6	2.35 ± 0.22^{cd}	4.04 ± 0.34^{c}	7.43 ± 0.19^{cd}	6.82 ± 0.24^{abc}	2.48 ± 0.02^{bcd}	10.63 ± 0.40^{bc}	33.74 ± 1.30^{c}	41.67 ± 2.18^{de}	44.20 ± 2.02^{d}
**Continuous N-lim**	N-resupply	Day 7	4.01 ± 0.53^{b}	6.05 ± 1.12^{b}	8.60 ± 0.64^{bc}	7.97 ± 0.84^{a}	2.89 ± 0.17^{abc}	12.89 ± 1.43^{a}	42.40 ± 4.06^{b}	66.10 ± 5.93^{b}	63.94 ± 6.83^{c}
	Steady state	Day 23-24	2.66 ± 0.24^{c}	3.37 ± 0.47^{cd}	8.08 ± 0.75^{bc}	5.64 ± 0.54^{bc}	2.58 ± 0.28^{bc}	8.86 ± 0.74^{c}	31.20 ± 2.63^{cd}	51.82 ± 5.43^{cd}	45.92 ± 3.87^{d}
**Batch N-starvation**		Day 28	2.33 ± 0.07^{cd}	2.94 ± 0.00^{cde}	7.63 ± 0.29^{cd}	5.92 ± 0.36^{bc}	2.70 ± 0.09^{abc}	9.24 ± 0.59^{c}	30.76 ± 0.05^{cd}	45.66 ± 2.36^{d}	41.58 ± 2.79^{de}
		Day 29	1.32 ± 0.12^{de}	2.09 ± 0.06^{de}	6.08 ± 0.52^{de}	5.33 ± 0.47^{c}	2.26 ± 0.01^{cd}	9.42 ± 0.54^{c}	26.51 ± 0.59^{d}	29.61 ± 2.08^{ef}	30.05 ± 2.20^{ef}
		Day 30	1.05 ± 0.16^{e}	1.94 ± 0.15^{e}	5.84 ± 0.77^{de}	5.56 ± 0.85^{bc}	1.85 ± 0.23^{d}	10.61 ± 0.14^{bc}	26.84 ± 2.26^{cd}	24.25 ± 2.70^{f}	28.07 ± 2.92^{f}
		Day 31	0.74 ± 0.12^{e}	1.72 ± 0.25^{e}	5.24 ± 0.88^{e}	5.22 ± 1.00^{c}	0.95 ± 0.16^{e}	10.85 ± 0.44^{abc}	24.72 ± 2.78^{d}	20.69 ± 2.93^{f}	26.03 ± 2.87^{f}
